# PTX3 Polymorphisms Influence Cytomegalovirus Reactivation After Stem-Cell Transplantation

**DOI:** 10.3389/fimmu.2019.00088

**Published:** 2019-01-31

**Authors:** Cláudia F. Campos, Luís Leite, Paulo Pereira, Carlos Pinho Vaz, Rosa Branca, Fernando Campilho, Fátima Freitas, Dário Ligeiro, António Marques, Egídio Torrado, Ricardo Silvestre, João F. Lacerda, António Campos Jr., Cristina Cunha, Agostinho Carvalho

**Affiliations:** ^1^Life and Health Sciences Research Institute (ICVS), School of Medicine, University of Minho, Braga, Portugal; ^2^ICVS/3B's-PT Government Associate Laboratory, Braga/Guimarães, Portugal; ^3^Serviço de Transplantação de Medula Óssea, Instituto Português de Oncologia do Porto, Porto, Portugal; ^4^Faculdade de Medicina de Lisboa, Instituto de Medicina Molecular, Lisbon, Portugal; ^5^Instituto Português do Sangue e Transplantação, IP, Porto, Portugal; ^6^Instituto Português do Sangue e Transplantação, IP, Lisbon, Portugal; ^7^Serviço de Imuno-Hemoterapia, Hospital de Braga, Braga, Portugal; ^8^Serviço de Hematologia e Transplantação de Medula, Hospital de Santa Maria, Lisbon, Portugal

**Keywords:** cytomegalovirus, stem-cell transplantation, PTX3, single nucleotide polymorphism, precision medicine, genomics

## Abstract

**Background:** Reactivation of latent human cytomegalovirus (CMV) in patients undergoing allogeneic stem-cell transplantation (HSCT) predisposes to several clinical complications and is therefore a major cause of morbidity and mortality. Although pentraxin-3 (PTX3) has been previously described to bind both human and murine CMV and mediate several host antiviral mechanisms, whether genetic variation in the *PTX3* locus influences the risk of CMV infection is currently unknown.

**Methods:** To dissect the contribution of genetic variation within *PTX3* to the development of CMV infection, we analyzed described loss-of-function variants at the *PTX3* locus in 394 recipients of HSCT and their corresponding donors and assessed the associated risk of CMV reactivation.

**Results:** We report that the donor, but not recipient, h2/h2 haplotype in *PTX3* increased the risk of CMV reactivation after 24 months following transplantation, with a significant effect on survival. Among recipients with h2/h2 donors, CMV seropositive patients as well as those receiving grafts from unrelated donors, regardless of the CMV serostatus, were more prone to develop viral reactivation after transplantation. Most importantly, the h2/h2 haplotype was demonstrated to display an influence toward risk of CMV reactivation comparable to that conferred by the unrelated status of the donor alone.

**Conclusions:** Our findings demonstrate the important contribution of genetic variation in donor *PTX3* to the risk of CMV reactivation in patients undergoing HSCT, highlighting a promising prognostic value of donor *PTX3* to predict risk of CMV reactivation in this clinical setting.

## Introduction

Human cytomegalovirus (CMV), a member of the *Herpesviridae* family, is a ubiquitous opportunistic pathogen that has intimately co-evolved with its human host and can establish latency after clearance of the primary infection (1). CMV asymptomatically infects the majority of the world's population (approximately 40–99%), with the highest seroprevalence in developing countries, and typically only leads to disease in the absence of an adequate cellular immunity (2). Asymptomatic long-term virus shedding in urine and saliva secretions usually marks the primary infection in healthy individuals (3). Throughout complex virus-host interactions, CMV evades a number of host pathways to enable its lifelong persistence, during which it may replicate chronically or reactivate from latency sporadically (4). In immunocompetent individuals, these reactivation events are tightly controlled by the immune system and rarely result in clinical presentation (5). However, it is becoming increasingly apparent that CMV may be associated with additional long-term health consequences due to its ability to establish lifelong persistence in critically ill patients (6, 7). Accordingly, reactivation of latent virus following allogeneic hematopoietic stem-cell transplantation (HSCT) has been increasingly associated with overt CMV disease, a major cause of morbidity and mortality in these patients. Despite important efforts centered in diagnostic and therapeutic advances, pre-emptive antiviral therapy is associated with significant myelotoxicity and impaired hematological reconstitution (8, 9), ultimately leading to other disease complications including superinfection by other viruses, bacteria and fungi, particularly *Aspergillus* species (10, 11).

The immune control of viral infections requires different components originating from both innate and adaptive arms of the immune system (12). Specifically, the innate immune system has evolved a multitude of unique antiviral humoral mechanisms through the participation of collectins, including surfactant protein (SP)-A and SP-D, and pentraxins (13–15). The long pentraxin-3 (PTX3) is a member of a superfamily of fluid-phase proteins, distinguished by their cyclic multimeric structure and the presence of a conserved amino acid signature in their C-terminal domain (16). In response to proinflammatory stimuli, PTX3 production is induced in a broad range of immune cells, including macrophages, dendritic cells and endothelial cells (17). Moreover, PTX3 is stored in the intracellular granules of neutrophils in a ready-made form and is rapidly released upon pathogen challenge or tissue damage, thereby covering a temporal window preceding PTX3 gene expression-dependent production. By acting as an ancestor of antibodies, PTX3 exerts a multifaceted nonredundant role in innate immunity against certain microbes by modulating complement activity and facilitating pathogen recognition by myeloid innate immune cells (18–20). As such, and although classic immunodeficiencies have not been linked to PTX3 deficiency (21), common polymorphisms have been disclosed as important risk factors across different infectious diseases, namely *Pseudomonas aeruginosa* colonization in cystic fibrosis patients (22), uropathogenic *Escherichia coli* infection (23), and invasive aspergillosis in recipients of HSCT (24, 25) and solid organ transplantation (26, 27), as well as patients with chronic obstructive pulmonary disease (28).

Despite a well-recognized role in innate host defense against selected bacteria and fungi, accumulating evidence also suggests the involvement of PTX3 in innate antiviral immunity (29). In fact, PTX3 has been described to act as a receptor decoy for the virus during CMV infection (30). Specifically, PTX3 was found to exert a protective role by binding both human and murine CMV, resulting in a reduced viral entry into permissive cells and resistance to *Aspergillus* superinfection, a mechanism entirely dependent on Toll-like receptors (TLRs) sensing pathways and activation of interferon (IFN) regulatory factor 3 (IRF3). Of note, the exogenous administration of PTX3 resulted in therapeutic efficacy against primary CMV infection and reactivation as well as *Aspergillus* superinfection in pre-clinical models of HSCT.

The compelling evidence that PTX3 is an effective mediator in preventing CMV infection and reactivation as well as subsequent superinfections pinpoints a potential role for PTX3 as a biomarker and therapeutic agent in viral infections and superinfections in the transplantation setting. However, the potential involvement of genetic variation in *PTX3* during CMV reactivation in at-high risk individuals has never been addressed. In this large genetic association study involving 394 eligible donor-recipient HSCT pairs, we provide crucial insights into the genetic contribution of PTX3 as a critical regulator of susceptibility to CMV infection. This information may ultimately lay the foundations toward risk stratification approaches aimed at a more effective and personalized management of CMV infection in this clinical setting.

## Materials and Methods

### Patients

A total of 460 hematological patients of European descent undergoing allogeneic HSCT at Instituto Português de Oncologia, Porto, and at Hospital de Santa Maria, Lisbon, between 2009 and 2015, were enrolled. Both donor and recipient DNA samples as well as patient-level data were available for 394 of these. The demographic and clinical characteristics of the patients are summarized in Table 1. One hundred and ninety-six cases of CMV infection and 198 uninfected controls were identified through pp65/pUL83 antigenemia assay (>1/100 pp65/pUL83 antigen-positive cells) and blood quantitative PCR for detection of viral DNA (>400 copies/mL) according to the recent revised standard criteria (31). All patients were monitored weekly for viral infection (reactivation or primary infection) with CMV until day +90 post-HSCT and subsequently every second week. In the event of increasing viral loads, pre-emptive therapy with valganciclovir was initiated. Approval for the study was obtained from the Ethics Subcommittee for Life and Health Sciences of the University of Minho, Portugal (125/014), the Ethics Committee for Health of the Instituto Português de Oncologia, Porto, Portugal (26/015), the Ethics Committee of the Lisbon Academic Medical Center, Portugal (632/014), and the National Commission for the Protection of Data, Portugal (1950/015). All participants provided written informed consent prior to transplantation in accordance with the Declaration of Helsinki.

**Table 1:** Demographic and transplant-related characteristics at baseline.

**Variable**	**Total (*n* = 394)**	**No CMV infection (*n* = 198)**	**CMV infection (*n* = 196)**	***P*-value^†^**
**AGE AT TRANSPLANTATION, NO (%)**				
≤20 years	75 (19.1)	42 (21.2)	33 (16.8)	0.37
21–40 years	103 (26.1)	54 (27.3)	49 (25.0)	
>40 years	216 (54.8)	102 (51.5)	114 (58.2)	
**GENDER, NO (%)**				
Female	169 (42.9)	90 (45.5)	79 (40.3)	0.29
Male	225 (57.1)	108 (54.5)	117 (59.7)	
**UNDERLYING DISEASE, NO. (%)**				
Acute leukemia	213 (54.1)	109 (55.1)	104 (53.1)	0.79
Chronic lymphoproliferative diseases	65 (16.5)	28 (14.1)	37 (18.9)	
Chronic myeloproliferative diseases	25 (6.3)	14 (7.1)	11 (5.6)	
Myelodysplastic/myeloproliferative diseases	57 (14.5)	28 (14.1)	29 (14.8)	
Aplastic anemia	19 (4.8)	10 (5.1)	9 (4.6)	
Others or unknown	15 (3.8)	9 (4.5)	6 (3.1)	
**TRANSPLANTATION TYPE, NO. (%)**				
Matched, related	180 (45.7)	106 (53.6)	74 (37.8)	0.009
Matched, unrelated	106 (26.9)	43 (21.7)	63 (32.1)	
Mismatched, related	6 (1.5)	4 (2.0)	2 (1.0)	
Mismatched, unrelated	102 (25.9)	45 (22.7)	57 (29.1)	
**GRAFT SOURCE, NO. (%)**				
Peripheral blood	324 (82.2)	166 (83.8)	158 (80.6)	0.10
Bone-marrow	62 (15.7)	31 (15.7)	31 (15.8)	
Cord blood	8 (2.0)	1 (0.5)	7 (3.6)	
**DISEASE STAGE, NO. (%)**				
First complete remission	219 (55.6)	120 (60.6)	99 (50.5)	0.13
Second or subsequent remission, or relapse	69 (17.5)	30 (15.2)	39 (19.9)	
Active disease	106 (26.9)	48 (24.2)	58 (29.6)	
**CONDITIONING REGIMEN, NO (%)**				
RIC	274 (69.5)	136 (68.7)	138 (70.4)	0.70
Myeloablative	120 (30.5)	62 (31.3)	58 (29.6)	
**CMV SEROSTATUS OF DONOR AND RECIPIENT, NO. (%)**				
D+/R+	270 (68.5)	133 (67.2)	137 (69.9)	< 0.0001
D–/R+	81 (20.6)	30 (15.2)	51 (26.0)	
D+/R–	23 (5.8)	18 (9.1)	5 (2.6)	
D–/R–	20 (5.1)	17 (8.6)	3 (1.5)	
**DURATION OF NEUTROPENIA, MEAN DAYS (RANGE)^‡^**				
	14 (5–39)	14 (6–39)	13 (5–35)	0.40
**ACUTE GVHD, NO. (%)**				
No GVHD or grades I–II	329 (83.5)	171 (86.4)	158 (80.6)	0.12
Grades III–IV	65 (16.5)	27 (13.6)	38 (19.4)	

† P-values were calculated by Fisher's exact probability t-test or Student's t-test for continuous variables, comparing the groups with and without CMV infection.

‡ *Neutropenia was defined as ≤0.5 × 10^9^ cells/L. RIC, reduced intensity conditioning; CMV, cytomegalovirus; D, donor; R, recipient; GVHD, graft-vs.-host-disease*.

### Single Nucleotide Polymorphism (SNP) Selection and Genotyping

Genetic variants in the *PTX3* gene analyzed in this study were selected based on their described functional consequences and previous association with infectious complications after HSCT (24). Genomic DNA was isolated from whole blood using the QIAmp DNA Blood Mini Kit according to the protocol supplied by the manufacturer (Qiagen, Hilden, Germany). Genotyping was performed using KASPar assays (LGC Genomics, Hertfordshire, United Kingdom) in an Applied Biosystem 7500 Fast Real-Time PCR system (Thermo Fisher Scientific, MA, United States), according to the manufacturer's instructions. Mean call rate for the SNP was >98%. Quality control for the genotyping results was achieved with negative controls and randomly selected samples with known genotypes.

### Statistical Analysis

The probability of CMV reactivation according to *PTX3* genotypes was determined using the cumulative incidence method and compared using the Gray's test (32). The cumulative incidence of CMV reactivation at 24 months after HSCT was computed with the *cmprsk* package for R version 2.10.1, with censoring of data at the date of last follow-up visit and relapse and death as competing events. All clinical and genetic variants achieving a *P* ≤ 0.15 in the univariate analysis were entered one by one in a pairwise model together and kept in the final model if they remained significant (*P* < 0.05). Multivariate analysis was performed using the subdistribution regression model of Fine and Gray. Overall survival, defined as the time from transplantation to death from any cause, was estimated with the use of the Kaplan-Meier method and evaluated according to *PTX3* genotypes with the use of the log-rank test. Power calculations were performed using the *powerSurvEpi* package 0.0.9 for R. Our sample size provided 80% power and a type I error below 5% for genetic variants with allele frequencies between 0.15 and 0.20 conferring a relative risk of 2.0.

## Results

### Genetic Variation in PTX3 Increases the Risk of CMV Reactivation After HSCT

The baseline demographic and transplantation characteristics of the enrolled HSCT patients are depicted in Table 1. No significant differences were observed among cases of CMV reactivation and control groups regarding the age at transplantation, gender, underlying hematological disease, graft source, disease stage at transplantation, conditioning regimen, development of acute graft-vs.-host disease (GVHD) and duration of neutropenia. However, an increased number of cases of CMV infection was detected among serologically positive recipients (R+), whereas recipients with negative CMV serostatus (R–) were instead more protected from viral reactivation (*P* < 0.0001). In addition, HSCT patients receiving grafts from unrelated donors were more prone to develop CMV infection, compared to those with related donors (*P* = 0.009).

**Figure 1:**
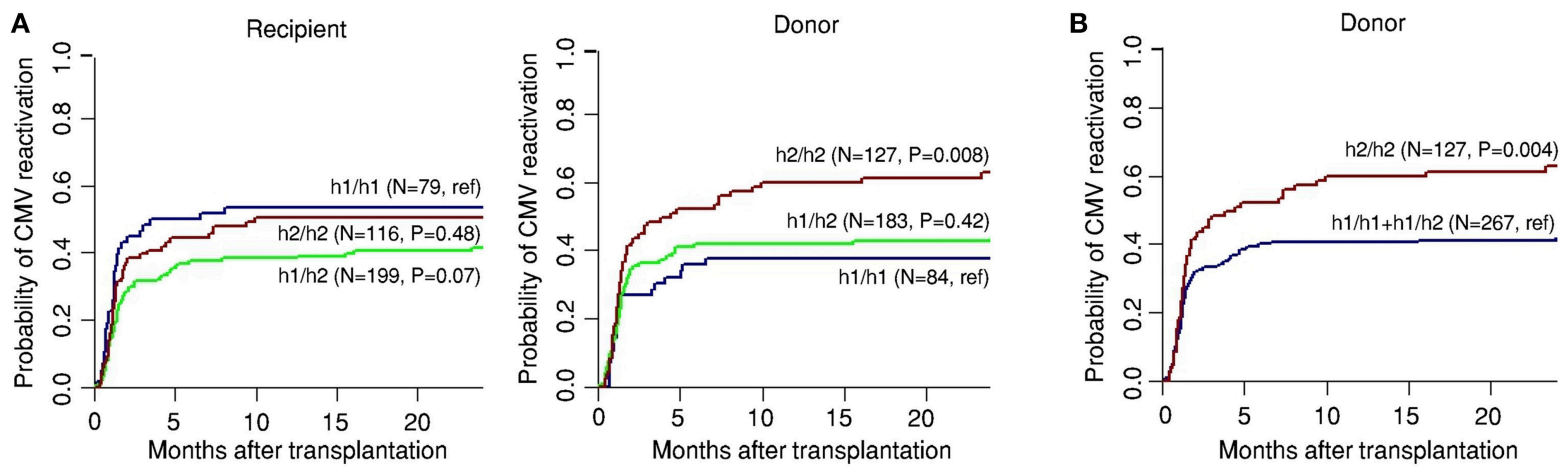
Genetic variation in donor *PTX3* increases the risk of CMV reactivation after HSCT. Genetic association study comprising 394 eligible HSCT recipients with available clinical data and corresponding donors. **(A)** Cumulative incidence of CMV reactivation after HSCT according to recipient and donor haplotypes in *PTX3*. **(B)** Cumulative incidence of CMV reactivation after HSCT according to donor haplotypes in *PTX3* following a recessive genetic model. Data were censored at 24 months, and relapse and death were considered competing events. *P*-values were calculated using Gray's test.

To investigate the relationship between genetic variation in *PTX3* and the susceptibility to CMV reactivation, the cumulative incidence of infection among transplant recipients was assessed according to recipient or donor genotypes at 24 months after HSCT. We found that donor, but not recipient, SNPs influenced the risk of CMV reactivation (Table 2). The cumulative incidence of CMV infection for donor rs2305619 was 59% for GG, 38% for AG and 36% for AA (*P* = 0.01), whereas for rs3816527, cumulative incidence of CMV infection was 55% for AA, 32% for AC and 37% for CC (*P* = 0.03). Haplotype analysis comprising rs2305619 and rs3816527 revealed that cumulative incidence of infection among patients with G-A/G-A [referred to as h2/h2 (24)] donors was 63% (*P* = 0.008) compared to 44 and 38% incidence observed for A-C/G-A (h1/h2) (*P* = 0.42) and A-C/A-C (h1/h1) donors, respectively (Figure 1A). In accordance with the results obtained for the individual SNPs, no significant influence of recipient haplotypes was observed on viral reactivation, with the cumulative incidence being 55% for h1/h1 (reference), 41% for h1/h2 (*P* = 0.07) and 50% for h2/h2 (*P* = 0.48). Other haplotypes were rare and were not included in the analysis (data not shown). The key contribution of the h2/h2 haplotype to the risk of infection was further illustrated upon modeling a recessive mode of inheritance (cumulative incidence of CMV reactivation, 63% for h2/h2 vs. 42% for h1/h1 and h1/h2 haplotypes combined; *P* = 0.004) (Figure 1B).

**Table 2:** Cumulative incidence of CMV reactivation according to recipient and donor *PTX3* genotypes, and association test results.

**RefSNP**	**Genotype(s)**	**Cumulative incidence of CMV reactivation at 24 Mo (%)**			
		**Recipient**	***P*-value**	**Donor**	***P*-value**
rs2305619	AA	52	0.12	36	0.01
	AG	38		38	
	GG	49		59	
rs3816527	AA	51	0.20	55	0.03
	AC	35		32	
	CC	52		37	

*CMV, cytomegalovirus; SNP, single nucleotide polymorphism; Mo, months. The P-values are for Gray's test using cumulative incidence analysis*.

In a multivariate model accounting for age, gender, donor relation and CMV serostatus, the donor h2/h2 haplotype was found to confer a 1.9-fold (95% confidence interval, 1.2–2.9; *P* = 0.004) increased risk of CMV reactivation after transplantation (Table 3). In addition, receiving grafts from either matched (*P* = 0.005) or mismatched (*P* = 0.004) unrelated donors also increased risk of infection, whereas CMV seronegative recipients displayed instead a decreased risk of infection (*P* = 0.01). Collectively, our results identify genetic variation in donor *PTX3* as a potentially critical risk factor influencing the susceptibility to CMV reactivation after HSCT.

**Table 3:** Multivariate analysis of the association of *PTX3* SNPs with the risk of CMV reactivation among transplant recipients.

**Genetic/clinical variables**	**Adjusted HR^†^ (95% CI)**	***P*-value**
Donor h2/h2 haplotype in *PTX3*	1.9 (1.2–2.9)	0.004
Matched unrelated donor	1.9 (1.2–3.0)	0.005
Mismatched unrelated donor	1.7 (1.2–2.5)	0.004
D+/R–	0.26 (0.09–0.74)	0.01

HR, hazard ratio; CI, confidence interval. Multivariate analyses were based on the subdistribution regression model of Fine and Gray.

† *Hazard ratios were adjusted for patient age and gender, type of transplantation, graft source, disease stage at transplantation, CMV serostatus of donor and recipient, and duration of neutropenia. Only the variables remaining significant after adjustment are shown*.

### The H2/H2 Haplotype Predisposes to CMV Infection Regardless of Viral Serostatus and Type of Donor

**Figure 2:**
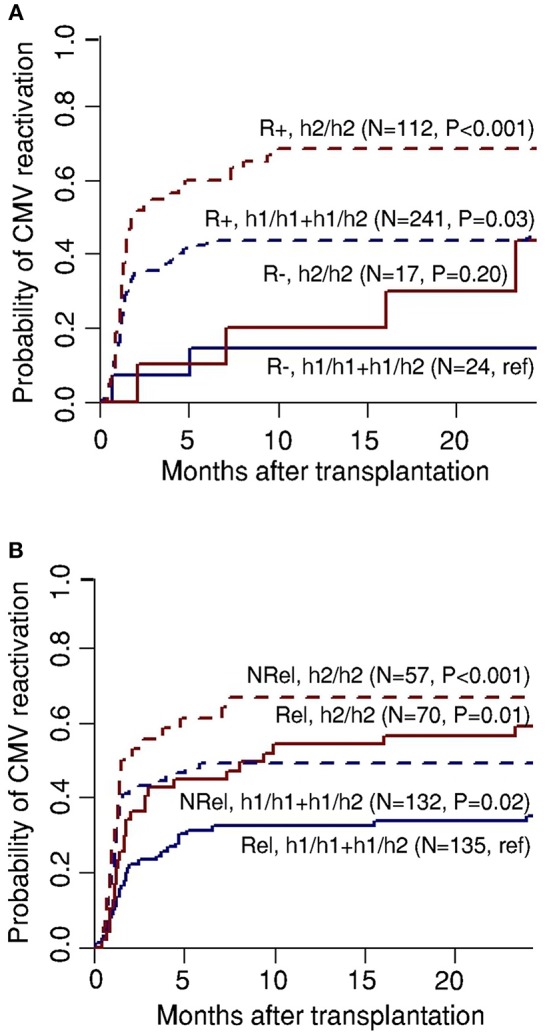
Recipient serostatus and donor relation synergize with genetic variation in *PTX3* toward risk of CMV reactivation. Genetic association study comprising 394 eligible HSCT recipients with available clinical data and corresponding donors. Cumulative incidence of CMV reactivation after HSCT according to donor haplotypes in *PTX3* in combination with **(A)** recipient serostatus (negative, R–, or positive, R+) or **(B)** type of donor (related, Rel, or NRel, unrelated). Data were censored at 24 months, and relapse and death were considered competing events. *P*-values were calculated using Gray's test.

Several studies have demonstrated that CMV seropositive patients retain a higher associated mortality in comparison with seronegative recipients who were transplanted from seronegative donors (33, 34). In light of our genetic data disclosing the h2/h2 haplotype as a critical risk factor for CMV reactivation after HSCT, we further stratified patients according to the recipient CMV serostatus. We observed that CMV seropositive patients (R+) carrying the risk-conferring haplotype in *PTX3* displayed the highest risk of CMV reactivation after HSCT (Figure 2A). Specifically, the cumulative incidence of CMV reactivation was 68% for R+ and h2/h2 (*P* < 0.001), 44% for R+ and h1/h1+h1/h2 (*P* = 0.03), 30% for R– and h2/h2 (*P* = 0.20), and 14% for R– and h1/h1+h1/h2 (reference) (Figure 2A). These results indicate that the h2/h2 haplotype promoted a further increased risk of CMV reactivation among both CMV seropositive and seronegative recipients.

Given that the type of donor is also a well-described pre-transplantation predictive factor for CMV reactivation (35), we analyzed our genetic results according to the type of donor (related, Rel, and unrelated, NRel). We found that recipients harboring the h2/h2 haplotype were at higher risk of CMV infection following HSCT, regardless of the type of transplant (Figure 2B). The cumulative incidence of viral reactivation was 66.7% for NRel and h2/h2 (*P* < 0.001), 56.3% for Rel and h2/h2 (*P* = 0.01), 44.4% for NRel and h1/h1+h1/h2 (*P* = 0.02), and 33% for Rel and h1/h1+h1/h2 (reference). Of note, the similar incidence of CMV reactivation observed in recipients transplanted from Rel and h2/h2 donors and those transplanted from NRel and h1/h1+h1/h2 donors support a comparable effect of genetic variation in *PTX3* and the type of donor toward CMV reactivation. Collectively, these results suggest that PTX3 may constitute an ideal candidate for antiviral prophylactic measures aimed at counteracting the onset of CMV reactivation in HSCT patients.

### The H2/H2 Haplotype Influences Post-transplant Mortality

**Figure 3:**
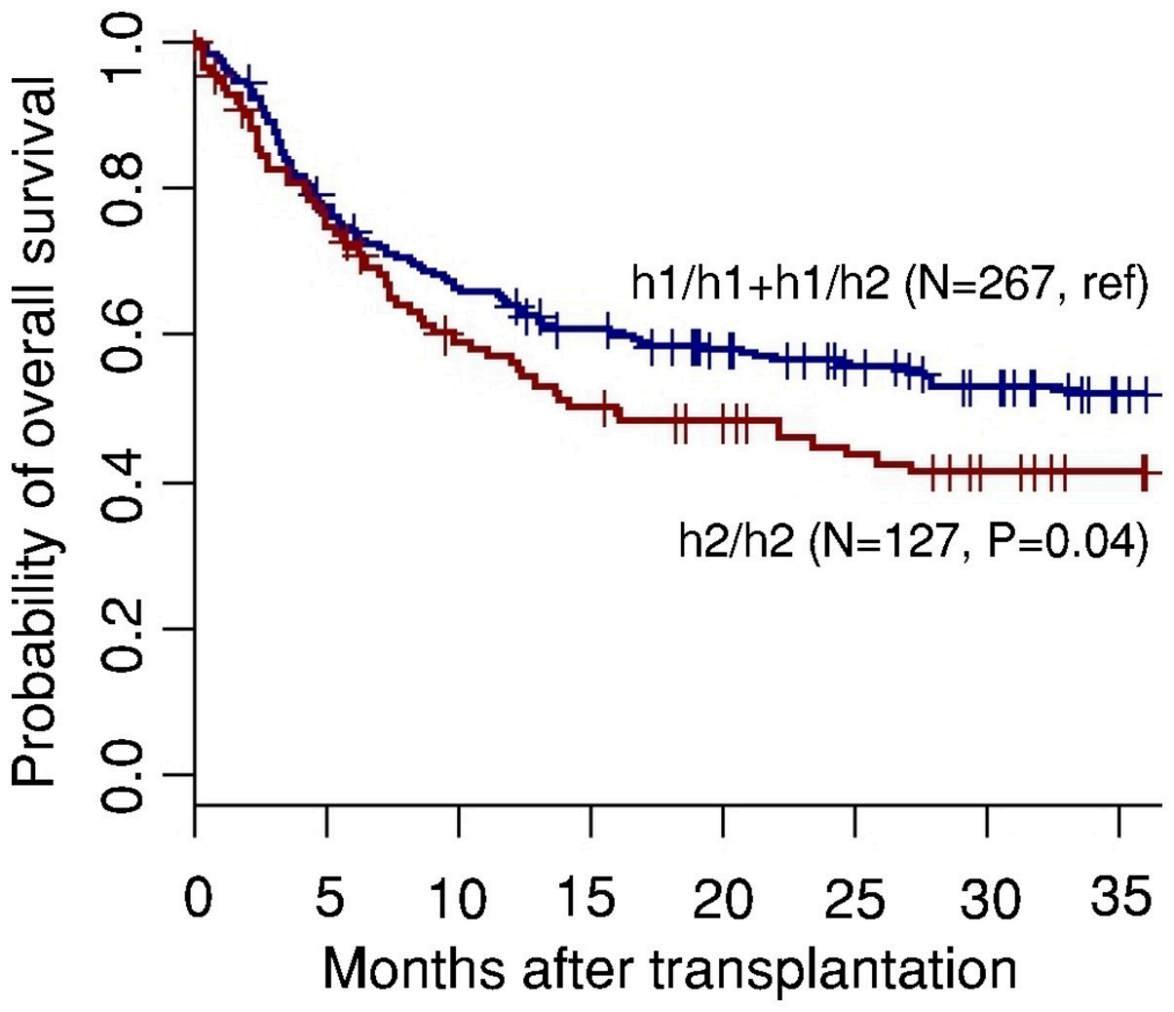
The donor h2/h2 haplotype in *PTX3* influences post-transplant survival of HSCT recipients. Overall survival (OS) according to donor haplotypes in *PTX3*. Data were censored at 36 months. *P*-values were calculated using the log-rank test.

In view of our findings highlighting the genetic variation in *PTX3* as promising predictive clinical candidate for CMV reactivation in HSCT patients, we next sought to investigate whether the donor h2/h2 haplotype influenced post-transplant mortality. The probability of overall survival was evaluated at 36 months following transplantation and estimated according to donor *PTX3* haplotypes. We observed that, consistent with the increased risk of CMV reactivation, the donor h2/h2 haplotype influenced the post-transplant survival of HSCT patients (Figure 3). The probability of survival decreased from 54% among patients who received transplants from either h1/h1 or h1/h2 donors to 45% among patients who received transplants from donors carrying the risk-conferring h2/h2 haplotype (*P* = 0.04). Taken together, these results highlight the potential role of genetic variation in *PTX3* as an important predictor of the risk of infection, but also the outcome of the patients.

## Discussion

Although remarkable advances in molecular virology and improvements in diagnostic methods and treatment regimen options have vastly enhanced our ability to manage CMV infection (36, 37), reactivation of latent virus remains one major cause of morbidity and mortality in patients undergoing HSCT (11). There is therefore a pressing demand for the development of novel prognostic markers for CMV reactivation aimed at supporting risk stratification measures and early diagnosis of infection. In this regard, the rs12979860 SNP upstream of the *IL28B* gene, known to be a critical factor associated with spontaneous clearance of hepatitis C (38), has been reported to influence the risk of CMV infection through the regulation of CMV-specific T-cell responses (39). Another study, despite failing to detect association, also revealed a contribution of the same *IL28B* variant to the levels of CMV DNAemia (40). In addition, SNPs in innate immunity genes, most notably TLR9 (41, 42), also appear to be important repositories of variability toward CMV infection across different studies. Collectively, these findings point to a strong genetic component in defining susceptibility to CMV reactivation after HSCT.

In this study, we have disclosed genetic variation in *PTX3* as an independent prognostic factor for CMV reactivation after transplantation, providing additional insights into human susceptibility to CMV infection. In addition, we determined a significant contribution of the donor h2/h2 haplotype to a poorer survival of HSCT recipients, similar to that previously reported among recipients of grafts from HLA-mismatched donors (24). This may reflect a key role of the h2/h2 haplotype in defining the outcome of HSCT patients by enhancing the risk of infectious complications. It remains to be assessed whether these genetic variants may also affect other non-infectious complications associated with HSCT. In this regard, it is worthwhile mentioning that plasma levels of PTX3 were increased at the onset of GVHD and were predictive of disease outcome (43), although the potential contribution of genetic variation in PTX3 to the risk and progression of GVHD remains to be assessed.

The integration of genetic markers into clinically valid processes to stratify the risk and progression of viral infection, and the efficacy of antiviral prophylaxis and therapy may represent a groundbreaking innovation for at-risk patients. In addition, the mechanistic involvement of PTX3 during CMV infection has been known for a long time (17, 44). By binding to CMV through sialic acids expressed on its glyosidic moiety, PTX3 was demonstrated to be effective in averting CMV infection and reactivation in selected *in vivo* and *in vitro* models of infection (30). Our genetic study associating a loss-of-function haplotype with the risk of CMV reactivation further supports the previously reported antiviral role of PTX3 and, considering its therapeutic potential in pre-clinical models (30), the administration of PTX3 could be envisaged as a promising immunotherapeutic approach to rescue the genetic deficiency in at-risk patients.

Although major advances have been accomplished regarding antiviral prophylactic strategies and preemptive therapy (9, 45), the donor and recipient CMV serological status still plays a major influence on the outcome of post-transplantation complications (46). Numerous investigations have demonstrated that CMV seronegative recipients transplanted from equally seronegative donors retain a reduced risk of transplant-related mortality, especially that caused by infections, in comparison with serologically positive recipients (47, 48). In line with the reported data, we found a significantly increased number of CMV reactivation cases among seropositive patients. Most importantly, our observation that R+ and h2/h2 recipients were at the highest risk of CMV reactivation highlights PTX3 as an ideal candidate for personalized medical interventions such as intensified diagnostics and targeted preemptive antiviral prophylaxis to prevent and counteract the onset of infection in specific subgroups of patients that are most at risk of viral reactivation.

Within the criteria for donor selection, the relation between donor and recipient constitutes one of the most relevant pre-transplantation predictors of CMV reactivation after transplantation (35, 49). In accordance, we found a significantly increased number of cases of CMV reactivation among patients with unrelated donors. Since the success of transplantation procedures hinges on the availability of suitable donors (50), our results suggest a pivotal role for PTX3 genetics as a pre-transplantation factor that could reshape current clinical approaches through the implementation of innovative risk stratification strategies that may involve the choice of alternative donors. Indeed, our results appear to indicate that selection of donors carrying the h2/h2 haplotype in *PTX3* may have a detrimental effect toward the reactivation of CMV after HSCT comparable to that conferred by the unrelated status of the donor alone.

Given its exploratory nature, our study presents certain limitations. The most important refer to the absence of definitive conclusions about the mechanism(s) through which genetic variation in donor *PTX3* influences the risk of developing CMV infection. Although it could presumably involve the transfer of CMV-specific lymphocytes, this hypothesis needs to be further explored. In addition, the low number of cases of overt CMV disease in our cohort precluded the appreciation of potential associations between severe viral disease and genetic variation in *PTX3*. Functional studies are ultimately required to understand how PTX3 is regulated in response and during CMV infection after HSCT, and what is the relative involvement of genetic variation in defining the levels of PTX3 and contributing to infection.

In conclusion, the evidence presented herein for a robust association between genetic variation in *PTX3* and CMV reactivation in patients undergoing HSCT highlights the significance of PTX3 as a promising marker for personalized medical intervention strategies, especially in the HSCT population, which may be particularly well-suited for genetically-targeted antiviral prophylaxis or enhanced diagnostic surveillance (51).

## Author Contributions

CFC, CC, JL, AnC, and AgC designed the study. LL, PP, CV, RB, FC, JL, and AnC oversaw patient recruitment, and collection of patient-level data. FF and DL coordinated the collection and storage of DNA samples. CFC and CC performed genotyping and the statistical analyses. CFC, ET, RS, CC, and AgC interpreted the data. All authors critically revised and approved the manuscript and are accountable for the accuracy and integrity of the work.

### Conflict of Interest Statement

The authors declare that the research was conducted in the absence of any commercial or financial relationships that could be construed as a potential conflict of interest.
